# Mutations of *folC* cause increased susceptibility to sulfamethoxazole in *Mycobacterium tuberculosis*

**DOI:** 10.1038/s41598-020-80213-4

**Published:** 2021-01-14

**Authors:** Ruiqi Wang, Kun Li, Jifang Yu, Jiaoyu Deng, Yaokai Chen

**Affiliations:** 1 Key Laboratory of Special Pathogens and Biosafety, Wuhan Institute of Virology, Center for Biosafety Mega-Science, Chinese Academy of Sciences, Wuhan, 430071 People’s Republic of China; 2 University of Chinese Academy of Sciences, Beijing, 100049 People’s Republic of China; 3 School of Life Sciences, Southwest University, Chongqing, People’s Republic of China; 4 Central Laboratory, Chongqing Public Health Medical Center, Chongqing, 400036 People’s Republic of China

**Keywords:** Genetics, Microbiology

## Abstract

Previous studies showed that mutation of *folC* caused decreased expression of the dihydropteroate synthase encoding gene *folP2* in *Mycobacterium tuberculosis* (*M. tuberculosis*). We speculated that mutation of *folC* in *M. tuberculosis* might affect the susceptibility to sulfamethoxazole (SMX). To prove this, 53 clinical isolates with *folC* mutations were selected and two *folC* mutants (I43A, I43T) were constructed based on *M. tuberculosis* H37Ra. The results showed that 42 of the 53 clinical isolates (79.2%) and the two lab-constructed *folC* mutants were more sensitive to SMX. To probe the mechanism by which *folC* mutations make *M. tuberculosis* more sensitive to SMX, *folP2* was deleted in H37Ra, and expression levels of *folP2* were compared between H37Ra and the two *folC* mutants. Although deletion of *folP2* resulted in increased susceptibility to SMX, no difference in *folP2* expression was observed. Furthermore, production levels of *para*-aminobenzoic acid (*p*ABA) were compared between the *folC* mutants and the wild-type strain, and results showed that *folC* mutation resulted in decreased production of *p*ABA. Taken together, we show that *folC* mutation leads to decreased production of *p*ABA in *M. tuberculosis* and thus affects its susceptibility to SMX, which broadens our understanding of mechanisms of susceptibilities to antifolates in this bacterium.

## Introduction

Tuberculosis (TB), which is caused by the single infectious agent *Mycobacterium tuberculosis* (*M. tuberculosis*), was known as the “white plague” in the preantibiotics era^1^. Today, even with various available antimicrobials, TB is still one of the deadliest diseases, claiming an estimated 1.5 million deaths worldwide in 2018^2^. The occurrence of multidrug-resistant (MDR) *M. tuberculosis*, which is co-resistant to rifampicin (RIF) and isoniazid (INH), complicates the control of TB^3,4^. It was estimated that there were globally 484,000 MDR/RIF-resistant (RR) TB cases in 2018^2^. The frequent increases in drug resistance reduce the number of drugs available to treat TB and renew the interest in old anti-TB drugs. Increasing our understanding of these old drugs with respect to action and resistance mechanisms is a faster and cheaper strategy compared with the research and development of new drugs.

Folate antagonists are one kind of renewed drugs that impose negative effects on different components of folate synthesis and metabolic pathways. The bacterial folate pathway is a valuable target for antimicrobial drug design due to the following two reasons. First, most of the microorganisms need to synthesize folates de novo, but mammals lack such a pathway and thus acquire folates from the diet; second, folate serves as a major one-carbon donor for multiple vital physiological processes, such as the biosynthesis of thymine, purines, and methionine^5^. Sulfonamides, the first antimicrobials used by humans to cure bacterial infections, compete with *para*-aminobenzoic acid (*p*ABA) and thus disrupt bacterial dihydropteroate biosynthesis by blocking the substrate required for dihydropteroate synthase^6^. Sulfamethoxazole (SMX) is one of the commonly used sulfonamides and is typically used in combination with trimethoprim (TMP), also known as cotrimoxazole (SXT), to treat respiratory and urinary tract infections^7,8^, or to prevent infection of *Pneumocystis carinii* in HIV-infected patients^9^. Afterward, clinical *M. tuberculosis* isolates were found to be susceptible to SXT regardless of their resistance to first-line anti-TB drugs^10^. However, other studies showed that SMX was active against *M. tuberculosis*, but TMP was not^11–13^. In addition, SMX was shown to have a synergistic effect with rifampicin and an additive effect with ethambutol in the treatment of *M. tuberculosis* isolates^12^. Recently, several reports showed that prophylaxis of HIV-positive patients with SXT reduced the risk of TB, indicating a potential role for SXT in the treatment of latent *M. tuberculosis* infection^14–17^.

*Para*-aminosalicylic acid (PAS), another folate antagonist, is the second antimicrobial used for the treatment of TB after introduction of streptomycin (STR)^18^. The combined usage of PAS and STR laid the foundation for the modern 6- to 9-month TB therapy^19^. Subsequently, because of gastrointestinal disturbance, PAS was replaced by ethambutol, a more tolerated drug. Although PAS has been used for more than 70 years, its mechanisms of action and resistance are still not fully understood. PAS is a structural analog of *p*ABA, and the addition of *p*ABA antagonizes the activity of PAS^20^. As a prodrug, PAS needs to be sequentially bio-activated by two enzymes involved in *M. tuberculosis* folate biosynthesis (dihydropteroate synthase and dihydrofolate synthase), and then the bio-activated metabolite of PAS inhibits dihydrofolate reductase (DHFR) and, eventually, bacterial growth^19^. Consequently, mutations of *folC* (the DHFR coding gene) impair PAS bio-activation and result in PAS resistance in *M. tuberculosis* clinical isolates^21,22^. Except for mutations in *folC*, mutations in the thymidylate synthase coding gene *thyA*^23^ and the riboflavin biosynthesis-related gene *ribD* also confer PAS resistance in *M. tuberculosis* clinical isolates^24^. Mutations in the above three genes could be identified in about two-thirds of the PAS-resistant clinical isolates^25^.

Recently, Liu et al. found that deletion of the *folP2* gene in *Mycobacterium smegmatis*, which encodes an ortholog of the dihydropteroate synthase^27^, resulted in increased susceptibility to SMX^26^. Recently, Wei et al. found that mutation of *folC* in *M. tuberculosis* clinical isolates led to a decreased expression of *folP2*^28^. We thus speculated that mutation of *folC* in *M. tuberculosis* might lead to decreased expression of *folP2* and hence affect the susceptibility to SMX. To test this hypothesis, we collected PAS-resistant clinical isolates and determined their susceptibilities to SMX. We constructed lab strains with *folC* point mutations and *folP2* deletion, and expression levels of *folP2* were compared between the *folC* mutants and their parental strain. In addition, *p*ABA production levels were compared between the *folC* mutants and their parental strain. In the present paper, we report our results.

## Results

### Characteristics of the selected clinical isolates

The characteristics of the isolates used in this study are summarized in Table 1. A total of 95 strains were selected. One of these is *M. tuberculosis* H37Rv (ATCC 27294), and the other 94 strains are randomly selected clinical isolates from different patients. Among the 94 clinical isolates, 80 (85.1%) were isolated from sputum. Concerning drug resistance, 68 (72.3%) and 19 (20.2%) isolates were defined as MDR and extensively drug-resistant (XDR), respectively. Isolates from male patients (63, 67%) were twofold more than those from female patients (31, 33%). The dominating age group was 25–44 years old, which accounted for 48 (51.1%) patients from whom *M. tuberculosis* strains were isolated. The detailed information of all isolates is listed in Table S2.

**Table 1:** Characteristics of clinical samples used in this study. Other^a^: samples did not belong to the above five types; Other^b^: isolates were resistant to one or several MDR- or XDR-relevant drugs, but were not defined as the types above.

Sample type	*n* (%)	Drug resistance profile	*n* (%)	Gender	*n* (%)
Sputum	80 (85.1%)	MDR	68 (72.3%)	Female	31 (33%)
Pleural fluid	1 (1.1%)	XDR	19 (20.2%)	Male	63 (67%)
Fiberoptic bronchoscopy lavage fluid	5 (5.3%)	Pan-susceptible	2 (2.1%)	Age	
Cerebrospinal fluid	1 (1.1%)	Other^b^	5 (5.3%)	≤ 24	18 (19.1%)
Pyogenic fluid	2 (2.1%)			25–44	48 (51.1%)
Other^a^	5 (5.3%)			45–64	24 (25.5%)
Total	94			≥ 65	4 (4.3%)

### *ThyA*, *ribD*, and *folC* mutational types in PAS-resistant isolates

Among the 95 strains selected in this study, five clinical isolates plus H37Rv were sensitive to PAS (< 1 μg/ml), and the remaining 89 (94.7%) clinical isolates were resistant to PAS (> 1 μg/ml) on Löwenstein–Jensen (L–J) solid medium (Table 2). The resistance to PAS on L–J medium was subsequently confirmed by the minimum inhibitory concentration (MIC) method on 7H10 medium (> 2 μg/ml) (Table 3). The *thyA*, *ribD,* and *folC* genes of all 95 isolates were sequenced. No mutations were observed in all three genes in H37Rv, five PAS-sensitive isolates, and two PAS-resistant isolates. The remaining 87 PAS-resistant isolates harbored mutations in at least one of these three genes. Most mutations are nucleotide substitutions, and only one base insertion was found (in *folC*) (Table 3). For mutations in *thyA*, nucleotide alterations occurred at positions 64, 127, 290, 329, 399, 440, 505, and 704. Mutation at position 399 (one isolate) resulted in a premature stop codon. The dominating mutation in *thyA* occurred at position 704 (15 of 23 isolates), changing arginine to proline. Mutations in *ribD* were uncommon, and only 10 isolates with *ribD* mutations were identified. Among them, seven isolates had mutations at the putative regulatory zone (nucleotide position − 12), and the remaining three isolates had mutations in the coding region (nucleotide position 745). For mutations in *folC*, the leading mutation in this gene was at nucleotide position 128 (33 of 53 isolates). Two isolates with mutations at nucleotide position 128 switched T to G, and the other 31 isolates converted T to C (Table 3).

**Table 2:** PAS resistance, mutational profiles, and distribution ratio of SMX-sensitive isolates. PAS: *para*-aminosalicylic acid; SMX: sulfamethoxazole; WT: wild-type.

PAS resistance profiles	*n* (%)	PAS mutational profiles	*n* (%)	Sensitive to SMX (*n*/*n*,%)
PAS-sensitive	5 (5.3%)	WT	7 (7.4%)	0/7 (0%)
PAS-resistant	89 (94.7%)	*thyA* single	23 (24.5%)	0/23 (0%)
		*ribD* single	10 (10.6%)	0/10 (0%)
		*folC* single	53 (56.4%)	42/53 (79.2%)
Total	94	*thyA* and *ribD* double	1 (1.1%)	0/1 (0%)

**Table 3:** Detailed information of mutations in *thyA*, *ribD*, and *folC* and PAS/SMX DST results of the 95 clinical isolates. *ID* identification, *NO* no mutations present, *MIC* minimal inhibitory concentration.

Sample information				MIC (L–J)	MIC (7H10)	
Mutational gene	Sample ID	Nucleotide alteration	Amino acid alteration	PAS (μg/ml)	PAS (μg/ml)	SMX (μg/ml)
Control	H37Rv	NO		< 1	< 0.1	50
	K6457	NO		< 1	< 0.1	50
	K8497	NO		< 1	0.2	50
	K6533	NO		< 1	0.2	50
	K5970	NO		< 1	< 1	50
	K8313	NO		< 1	0.2	50
	K3270	NO		> 1	> 16	50
	E260	NO		> 1	> 16	50
*thyA*	K8902	64A>C	Thr22Pro	> 1	> 16	50
	K6328	127C>T	Pro43Ser	> 1	> 16	50
	K6487	290A>G	Gln97Arg	> 1	> 16	50
	K6407	329A>G	Asp110Gly	> 1	8	50
	K3543	399G>A	Trp133Stop	> 1	> 16	50
	K4287	440A>G	His147Arg	> 1	> 16	50
	K5819	440A>G	His147Arg	> 1	> 16	100
	E945	505G>A	Asp169Asn	> 1	> 16	50
	F461	704G>C	Arg235Pro	> 1	> 16	50
	K5999	704G>C	Arg235Pro	> 1	> 16	50
	K3229	704G>C	Arg235Pro	> 1	> 16	50
	K4208	704G>C	Arg235Pro	> 1	> 16	50
	K6079	704G>C	Arg235Pro	> 1	> 16	50
	E531	704G>C	Arg235Pro	> 1	> 16	50
	F330	704G>C	Arg235Pro	> 1	> 16	50
	E940	704G>C	Arg235Pro	> 1	> 16	> 100
	F462	704G>C	Arg235Pro	> 1	> 16	50
	K9133	704G>C	Arg235Pro	> 1	> 16	50
	E576	704G>C	Arg235Pro	> 1	> 16	50
	K2481	704G>C	Arg235Pro	> 1	> 16	50
	K8646	704G>C	Arg235Pro	> 1	> 16	50
	K3211	704G>C	Arg235Pro	> 1	> 16	50
	K5514	704G>C	Arg235Pro	> 1	> 16	50
*ribD*	K5874	− 12G>A		> 1	16	50
	K8941	− 12G>A		> 1	4	100
	K7234	− 12G>A		> 1	> 2	50
	K8433	− 12G>A		> 1	> 2	50
	E944	− 12G>A		> 1	8	50
	K2614	− 12G>A		> 1	> 2	50
	K4239	− 12G>A		> 1	> 2	50
	K4160	745G>A	Gly249Ser	> 1	> 2	50
	K3491	745G>A	Gly249Ser	> 1	> 2	50
	K4854	745G>A	Gly249Ser	> 1	> 2	50
*thyA*; *ribD*	F241	704G>T; 273G>A	Arg235Leu; Glu91Glu	> 1	4	100
*folC*	K6599	118G>A	Glu40Lys	> 1	16	50
	KA792	119A>G	Glu40Gly	> 1	> 2	10
	K3283	119A>G	Glu40Gly	> 1	2	20
	E958	119A>G	Glu40Gly	> 1	16	10
	F508	128T>G	Ile43Ser	> 1	> 2	< 5
	K6640	128T>G	Ile43Ser	> 1	> 2	5
	E941	128T>C	Ile43Thr	> 1	> 16	50
	K4279	128T>C	Ile43Thr	> 1	> 16	50
	K6545	128T>C	Ile43Thr	> 1	> 16	50
	K8315	128T>C	Ile43Thr	> 1	> 16	50
	E578	128T>C	Ile43Thr	> 1	> 16	50
	K8301	128T>C	Ile43Thr	> 1	> 16	20
	K3361	128T> C	Ile43Thr	> 1	> 16	20
	E903	128T>C	Ile43Thr	> 1	> 16	20
	K2543	128T>C	Ile43Thr	> 1	> 16	10
	K4913	128T>C	Ile43Thr	> 1	4	20
	K7601	128T>C	Ile43Thr	> 1	2	< 5
	K4385	128T>C	Ile43Thr	> 1	16	20
	F122	128T>C	Ile43Thr	> 1	> 16	50
	K7678	128T>C	Ile43Thr	> 1	16	20
	K4603	128T>C	Ile43Thr	> 1	16	10
	E930	128T>C	Ile43Thr	> 1	> 16	50
	K3183	128T>C	Ile43Thr	> 1	> 2	20
	K6361	128T>C	Ile43Thr	> 1	> 2	20
	KA387	128T>C	Ile43Thr	> 1	> 2	20
	KA045	128T>C	Ile43Thr	> 1	> 2	20
	KA156	128T>C	Ile43Thr	> 1	> 2	10
	KA636	128T>C	Ile43Thr	> 1	> 2	20
	K7917	128T>C	Ile43Thr	> 1	> 2	20
	K7040	128T>C	Ile43Thr	> 1	8	20
	K7805	128T>C	Ile43Thr	> 1	4	5
	K7979	128T>C	Ile43Thr	> 1	> 16	20
	F036	128T>C	Ile43Thr	> 1	8	5
	K3459	128T>C	Ile43Thr	> 1	> 16	20
	K5236	128T>C	Ile43Thr	> 1	8	10
	K4354	128T>C	Ile43Thr	> 1	> 16	50
	K5366	128T>C	Ile43Thr	> 1	> 16	20
	E285	127A>G; 128T>C; 129C>T	Ile43Val; Ile43Thr; Ile43Ile	> 1	> 16	50
	K4303	131A>G; 136A>C	Asp44Gly; Ser46Arg	> 1	16	50
	F970	145C>T	Arg49Trp	> 1	> 2	20
	F899	146G>A	Arg49Gln	> 1	2	20
	F077	448A>G	Ser150Gly	> 1	16	20
	K5303	448A>G	Ser150Gly	> 1	4	20
	F375	448A>G	Ser150Gly	> 1	> 2	10
	KA187	448A>G	Ser150Gly	> 1	> 2	10
	K6842	448A>G	Ser150Gly	> 1	> 16	20
	K6722	458A>C	Glu153Ala	> 1	> 2	20
	KA779	458A>C	Glu153Ala	> 1	> 2	< 5
	KA712	458A>C	Glu153Ala	> 1	> 2	10
	KA391	458A>C	Glu153Ala	> 1	> 2	5
	KA440	458A>C	Glu153Ala	> 1	> 2	10
	G216	458A>G	Glu153Gly	> 1	> 2	5
	K9669	529_530insG	Frame shift	> 1	16	20

### Most PAS-resistant clinical isolates with *folC* mutations were sensitive to SMX

To test SMX susceptibilities, H37Rv, five PAS-sensitive strains, and two PAS-resistant strains with no mutation in *thyA*, *ribD,* and *folC* were used as control strains. SMX MICs of these eight strains were all determined to be 50 μg/ml (Table 3). PAS-resistant strains with either *thyA* or *ribD* mutations were not more sensitive to SMX. On the contrary, among the 53 PAS-resistant strains with *folC* mutations, 42 (79.2%) were more sensitive to SMX compared with the eight control strains (Tables 2 and 3).

### *M. tuberculosis *H37Ra Δ*folP2*, *M. tuberculosis *H37Ra Δ*folC* pMV361::*folC*(I43T), and *M. tuberculosis *H37Ra Δ*folC* pMV361::*folC*(I43A) were more sensitive to SMX than the parental strain

**Figure 1:**
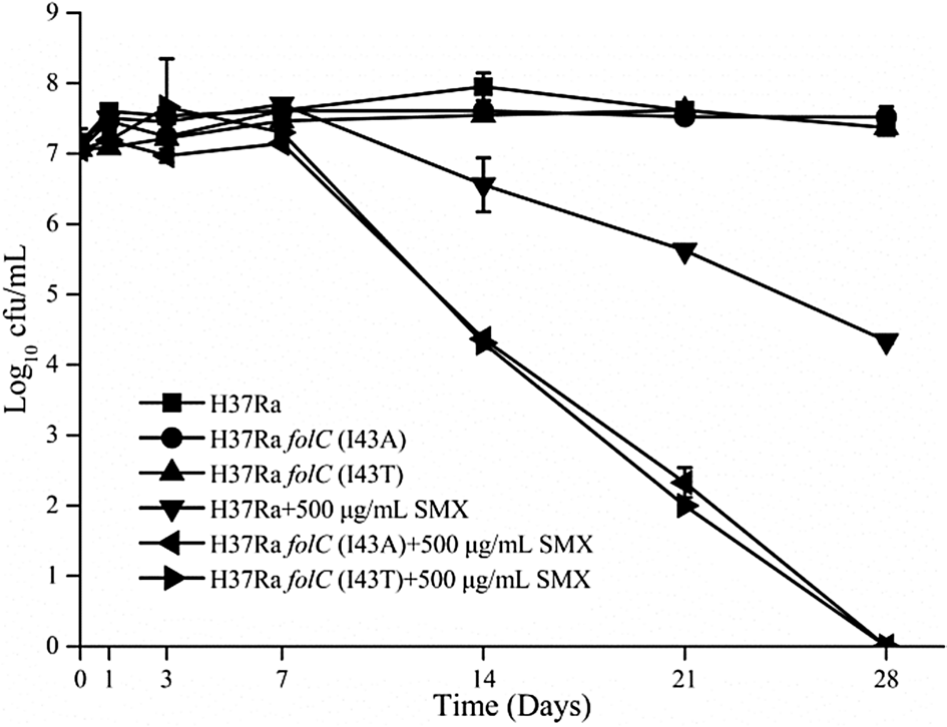
Killing curves of different *M. tuberculosis* strains upon SMX treatment. Liquid medium (7H9 + 10% OADC) was used. Experiments were performed in three biological replicates. Standard deviations are indicated by error bars.

SMX susceptibilities were tested in two lab-constructed *folC* mutants^21^ and *M. tuberculosis* H37Ra Δ*folP2*. The results showed that, compared with their parental strain, all three mutants were more sensitive to SMX (Table 4). In addition, the results of the killing curve assay showed that the two *folC* mutants were more susceptible to SMX than their parental strain (Fig. 1).

**Table 4:** PAS and SMX susceptibilities of different strains derived from *M. tuberculosis* H37Ra.

Strain	Description	MIC to PAS (μg/ml)	MIC to SMX (μg/ml)
H37Ra pMV261	H37Ra transformed with pMV261	0.04	100
H37Ra Δ*folP2*	Specialized transduction of strain H37Ra with phAES*folP2*_Ra_	0.08	50
H37Ra Δ*folC* pMV361::*folC*I43T	H37Ra carrying pMV361-*folC*I43T, chromosomal copy of *folC* deleted	10.24	12.5
H37Ra Δ*folC* pMV361::*folC*I43A	H37Ra carrying pMV361-*folC*I43A, chromosomal copy of *folC* deleted	10.24	12.5

### *FolC* mutations did not affect the expression of *folP2*

**Figure 2:**
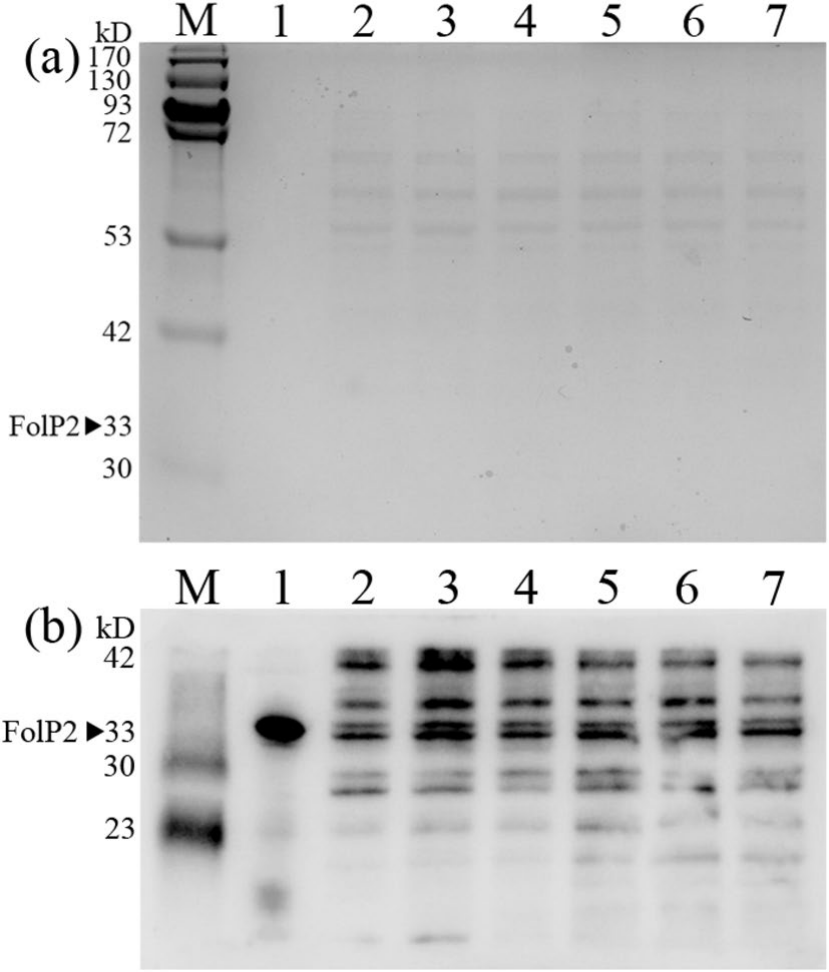
Comparison of the protein levels of FolP2 during the exponential phase in H37Ra, H37Ra *folC*(I43A), and H37Ra *folC*(I43T) strains, as indicated by western blot. Experiments were repeated at least three times, and representative results are shown. (**a**) Total protein (20 μg per lane) was separated by SDS-PAGE and stained by Coomassie brilliant blue. Lane M, the prestained protein marker. Lane 1, purified FolP2 protein. Lanes 2 and 3, total protein of H37Ra. Lanes 4 and 5, total protein of H37Ra *folC*(I43A). Lanes 6 and 7, total protein of H37Ra *folC*(I43T). (**b**) Western blot analysis of total protein immunoblotted with mouse antiserum with anti-FolP2. Lane 1, anti-FolP2 visualizing the purified FolP2 protein. Lanes 2 and 3, anti-FolP2 visualizing the total protein of H37Ra. Lanes 4 and 5, anti-FolP2 visualizing the total protein of H37Ra *folC*(I43A). Lanes 6 and 7, anti-FolP2 visualizing the total protein of H37Ra *folC*(I43T).

**Figure 3:**
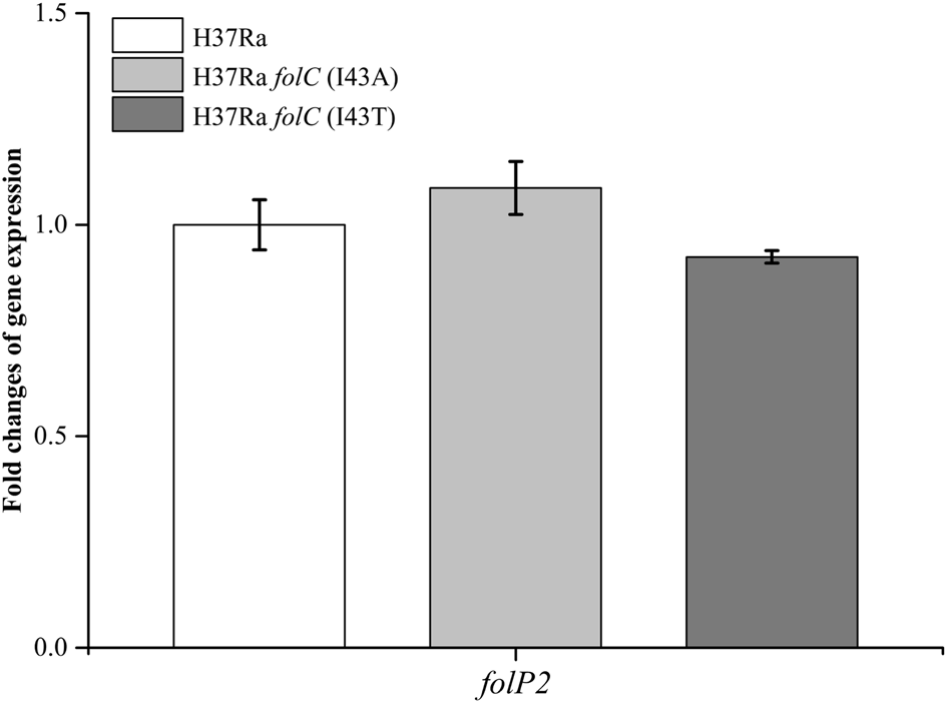
Comparison of the transcription levels of the gene *folP2* during the exponential phase in wild-type H37Ra and H37Ra *folC* point mutant strains (I43A, I43T) by quantitative real-time PCR. mRNA expression levels were normalized against *sigA* as an endogenous control. Data are representative of three experiments. Standard deviations are indicated by error bars.

**Figure 4:**
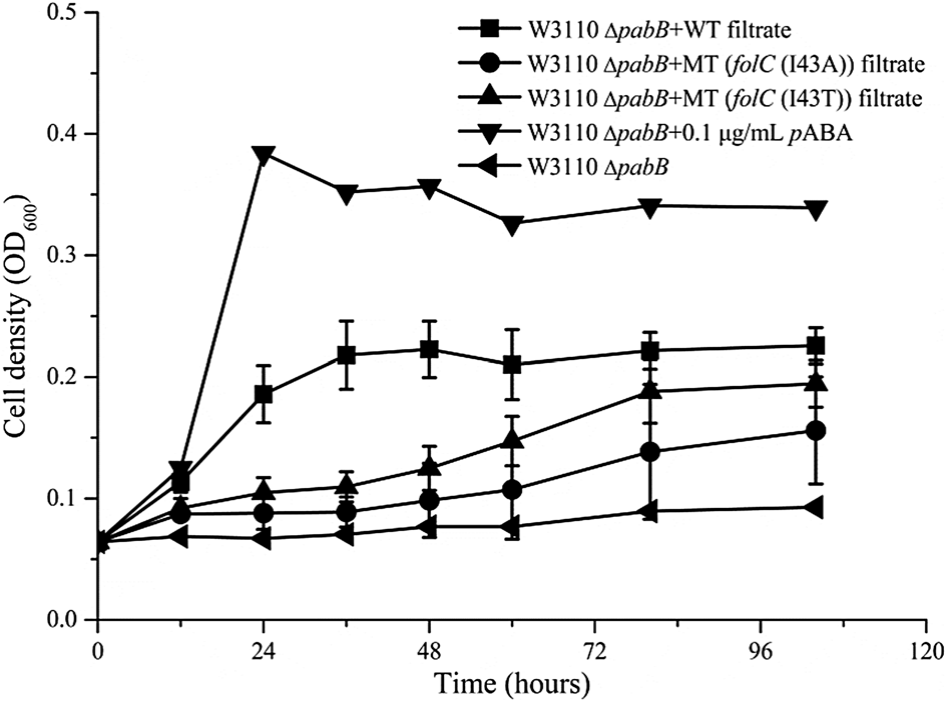
Mutation of *folC* caused decreased production of *p*ABA. Growth curves of *E. coli* W3110 Δ*pabB* were measured in the presence of *p*ABA and culture filtrates from wild-type H37Ra (WT-filtrate) and the two H37Ra *folC* mutant strains (MT-filtrates). Experiments were performed in triplicate. Standard deviations are indicated by error bars.

Results of the quantitative real-time PCR assay showed that no difference in the *folP2* expression level could be observed between *M. tuberculosis* H37Ra and the two *folC* mutants, which was confirmed by subsequent western blot analysis (Figs. 2 and 3). To determine production levels of *p*ABA in the three different H37Ra strains, an *Escherichia coli* (*E. coli*) W3110 Δ*pabB* mutant was used as previously described^29^. When *E. coli* W3110 Δ*pabB* was cultured in E minimal medium plus culture filtrates from different H37Ra strains, there was obviously less growth when culture filtrates from the two *folC* mutants were used (Fig. 4), suggesting a decreased production of *p*ABA.

## Discussion

A huge obstacle in defeating TB is the increasing resistance to scanty anti-TB drugs. This predicament reinforced our interest in exploring the usable medicines in the approved drugs treasure chest. Antifolates, due to their toxicity in folate biosynthesis, which is vital and greatly different between human cells and bacteria, are potentially important choices to treat MDR TB. In fact, after being put on the shelf for several decades, PAS was reintroduced in the 1990s for treating MDR TB^30^. However, resistance to PAS appeared in clinical *M. tuberculosis* isolates in the early 2000s. Although SXT has not been tried to treat TB clinically, attempts have been made to make it an option for treating drug-resistant TB^31–34^. In this context, better use of antifolates against TB has gained importance, and further in-depth studies on the mechanisms of susceptibility and resistance of antifolates are required.

Very recently, researchers found that in PAS-resistant clinical isolates of *M. tuberculosis* with *folC* mutation, protein expression levels of *folP2* were significantly lower than in strains with no *folC* mutation^28^. Though FolP2 of *M. tuberculosis* has been predicted to be defective in dihydropteroate synthesis activity^27^, deletion of the *folP2* gene in *M. smegmatis* resulted in increased susceptibility to SMX^26^. We also knocked out the *folP2* gene in *M. tuberculosis* H37Ra, and found that the resulting Δ*folP2* mutant was more sensitive to SMX. Therefore, we speculated that decreased expression of *folP2* caused by *folC* mutation might lead to increased susceptibility to SMX.

As expected, most of the PAS-resistant isolates with mutations in the *folC* gene showed increased susceptibility to SMX. In addition, two lab-constructed *folC* mutants were also more sensitive to SMX. These data suggest that mutation of *folC* did lead to increased susceptibility to SMX in *M. tuberculosis*.

We noted that 11/53 (20.8%) PAS-resistant isolates with *folC* mutations were not more sensitive to SMX, including isolates with I43A or I43T mutations. All PAS-resistant isolates used in this study were MDR/XDR clinical isolates obtained from different patients, suggesting the complexity of the genetic backgrounds of those isolates. A previous study using transmission electron microscopy showed that the cell walls of MDR and XDR strains were thicker than those of the susceptible *M. tuberculosis* isolates^35^, indicating another possible explanation.

However, subsequent quantitative real-time PCR and western blot analysis showed that *folC* mutations did not affect the expression of *folP2*, suggesting that the increased susceptibilities of the *folC* mutants to SMX were not caused by decreased expression of *folP2*.

With respect to the mechanisms of action, SMX shares one thing with PAS: both drugs compete with *p*ABA. As a result, deficiency in *p*ABA biosynthesis usually leads to increased susceptibility to both drugs^29,36^. We thus speculated that mutation of *folC* might lead to decreased production of *p*ABA and hence affect the susceptibility to SMX. Subsequent comparison of *p*ABA production levels between the two lab-constructed *folC* mutants and their parental strain confirmed this hypothesis.

To the best of our knowledge, this is the first study on the interaction of the two antifolates in the treatment of MDR or XDR TB clinical isolates. We found that the *folC* mutation in *M. tuberculosis* leads to decreased production of *p*ABA and hence increases sensitivity to SMX. Since our previous data showed that a small proportion (~ 9%) of the MDR strains had a mutation in *folC*^21^, it would be interesting to test the efficacy of SMX or SXT against those MDR strains in vivo. The regulation of folate metabolism is still obscure in *M. tuberculosis*, and our pioneering observations provide new evidence to guide future research.

## Materials and methods

### *M. tuberculosis* clinical isolates and drug susceptibility testing

Clinical samples (Table 1), such as sputum and pleural fluid, were pretreated and cultured in a BACTEC MGIT 960 system (Becton Dickinson, Sparks, MD, USA), according to the manufacturer’s instructions^37^. Positive cultures were subjected to (i) mycobacterium species identification and (ii) drug susceptibility testing (DST) using the proportion method on L–J solid medium (Encode, Zhuhai, China), where MDR was defined as resistance to at least isoniazid and rifampicin, and XDR was defined as MDR plus resistance to any fluoroquinolones (ofloxacin, levofloxacin or moxifloxacin) and at least one injectable drug (amikacin or capreomycin). Mycobacterium species identification was performed based on (i) sequence polymorphisms in 16S rRNA, *hsp65*, and *rpoB*^38^ and (ii) the results of bacterial growth on L–J solid medium containing 5 μg/ml TCH and 500 μg/ml PNB. MDR and XDR isolates were chosen for preliminary screening of PAS resistance using the same method and medium as described above. For DSTs of PAS or SMX on Middlebrook 7H10 solid medium (Difco, Becton Dickinson, Sparks, MD, USA), the two drugs (purchased from Merck, Darmstadt, Germany) were dissolved in deionized water and dimethyl sulfoxide (Merck) at concentrations of 10 mg/ml and 60 mg/ml, respectively. The drugs were frozen at − 20 °C after sterilization. The critical concentration of PAS was 1 μg/ml on L–J medium and 2 μg/ml on 7H10 medium according to policy guidelines on DST for second-line anti-TB drugs^39^. Isolates were frozen in 25% glycerol at − 70 °C until use. Our study was conducted in accordance with the Declaration of Helsinki. The institutional review board of Chongqing Public Health Medical Center approved this study and waived the requirement for written informed consent. The institutional review board waived the need for informed consent because all patients’ data were analyzed in anonymity and no additional informed consent was required.

### *ThyA*, *ribD*, and *folC* amplification and sequence analysis of PAS-resistant clinical isolates

The entire open reading frame flanking 150 bp of the upstream putative regulatory sequence of each of the three genes was amplified using the following primers: for *folC* amplification, the forward primer was *folC*-F (5′-CGGTCAGCAGTATCAACAGCACGGC-3′) and the reverse primer was *folC*-R (5′-CGCCGCCTGGAAAAGGAGTTGG-3′); for *thyA* amplification, the forward primer was *thyA*-F (5′-TGATCTCCCGGAAATGCGCCTGGT-3′) and the reverse primer was *thyA*-R (5′-GGTTTTCGGCATGGCCTCCGTTGTA-3′); and for *ribD* amplification, the forward primer was *ribD*-F (5′-CCGGCAAAAGTCCTGGCACGCCACG-3′) and the reverse primer was *ribD*-R (5′-GTTCTTGGGTGCGGCGAGCGGTGGT-3′). The primers were designed according to the *M. tuberculosis* reference sequence (Gene Bank accession number AL123456.3). For *thyA* amplification, an S1000 Thermal cycler (BIO-RAD, Hercules, CA, USA) was used with the following PCR program: denaturation at 98 °C for 10 min, followed by thirty cycles of denaturation at 98 °C for 15 s, annealing at 63.2 °C for 15 s, and extension at 72 °C for 30 s, and a final extension at 72 °C for 5 min. Amplification of *ribD* and *folC* was carried out following the same protocol, except the annealing temperatures were 66.5 °C for *ribD* and 64.3 °C for *folC*.

Agarose gel electrophoresis was performed, and the DNA was purified with an EZNA bacterial DNA kit (Omega Bio-Tek, Norcross, GA, USA) according to the manufacturer’s instructions. The purified DNA products were sequenced in an automatic DNA sequencer (model 3730XL, ABI, Foster City, CA, USA) with the same primers used for the PCR amplifications. Sequencing results were compared with reference sequences using nucleotide BLAST (https://blast.ncbi.nlm.nih.gov/Blast.cgi), and polymorphisms were depicted according to Sequence Variant Nomenclature (http://varnomen.hgvs.org/).

### Construction of H37Ra Δ*folP2*

A modified strategy for specialized transduction was used to construct the *M. tuberculosis* H37Ra Δ*folP2* mutant^40^. Genomic regions flanking *folP2*, 824 bp upstream (a region containing *MRA_1215*) and 827 bp downstream (a region containing *MRA_1217*), were amplified by PCR. The primers used for the amplification of the upstream region of *folP2* were *folP2*koLFP and *folP2*koLRP, and those for the downstream region were *folP2*koRFP and *folP2*koRRP. The recombinant plasmid p0004s-L + R was constructed by inserting the Van91I-digested PCR products into Van91I-digested plasmid p0004s. Then, p0004s-L + R was digested with PacI and ligated into the PacI-digested shuttle phasmid vector phAE159. After ligation, the recombinant cosmid phAE159-p0004s-L + R was transduced into *E. coli* HB101 in an in vitro λ-packaging reaction (MaxPlax Lambda Packaging Extracts, Epicentre Biotechnologies, Madison, WI, USA). The phasmid DNA prepared from confirmed selected hygromycin-resistant transductant was electroporated into *M. smegmatis* mc^2^155 to generate the specialized transducing phage. As described in a previous study^40^, the transducing phage at the most efficient titer was used to infect H37Ra at a multiplicity of infection of 10. Successful transduction of H37Ra was confirmed by comparing the size of the PCR-amplified products of hygromycin-resistant colonies with the wild-type H37Ra using primers *folP2*LYZ and *folP2*RYZ (Table S1).

### Drug susceptibility testing of *M. tuberculosis *H37Ra Δ*folP2*, *M. tuberculosis *H37Ra Δ*folC* pMV361::*folC*(I43T), and *M. tuberculosis *H37Ra Δ*folC* pMV361::*folC*(I43A)

DST was performed as previously described^41^. Briefly, *M. tuberculosis* H37Ra Δ*folP2*, *M. tuberculosis* H37Ra Δ*folC* pMV361::*folC*(I43T), and *M. tuberculosis* H37Ra Δ*folC* pMV361::*folC*(I43A) were grown at 37 °C in Middlebrook 7H9 medium plus 10% oleic acid–albumin–dextrose–catalase (OADC) to the mid-log phase (OD_600_ 0.4–0.8). Bacterial cultures were centrifuged, and cell pellets were collected and washed twice with 7H9. After tenfold serial dilution, 10 μL of diluted bacterial cells (approximately 10^5^ CFU/ml) were plated on Middlebrook 7H10 medium plus 10% OADC containing various concentrations of PAS (0, 0.0025, 0.005, 0.01, 0.02, 0.04, 0.08, 0.16, 0.32, 0.64, 1.28, 2.56, 5.12, and 10.24 μg/ml) and SMX (0, 0.78125, 1.5625, 3.125, 6.25, 12.5, 25, 50, 100, and 200 μg/ml). The plates were incubated at 37 °C for 3 weeks to determine the MIC values. The MIC was defined as the lowest required concentration of antibiotics to inhibit the growth of 99% of the bacterial CFUs.

### Quantitative real-time PCR assays

An RNeasy Mini kit (Qiagen, Germany) was used to extract total RNA, and a ReverTra Ace qPCR kit (TOYOBO, Osaka, Japan) was used to synthesize cDNA. All kits were used according to the manufacturers’ instructions. Gene expression levels were quantified using quantitative real-time PCR analysis on a 7900 HT Sequence Detection System (ABI, Foster City, CA, USA) with ABI Power SYBR Green PCR Master Mix (ABI). mRNA expression levels were normalized to *sigA* as an endogenous control. Primers for *folP2* amplification (*folP2*-qRT-L and *folP2*-qRT-R) were synthesized by Sangon Biotech Co., Ltd. (Shanghai, China) (Table S1).

### Purification of recombinant histidine-tagged FolP2

The *folP2* gene of *M. tuberculosis* H37Ra was amplified using primers M *folP2*-L and M *folP2*-R (Table S1) and subsequently cloned into pET28a to obtain the pET28a::*folP2* recombinant plasmid. The recombinant plasmid was verified and transformed into *E. coli* BL21 (DE3). The recombinant strain *E. coli* BL21 pET28a::*folP2* was grown at 37 °C in liquid LB medium to the mid-log phase (OD_600_ 0.4–0.8), 0.2 mM isopropyl-β-D-thiogalactopyranoside was added, and the cells were incubated at 16 °C for another 20 h. After induction, the bacterial cells were centrifuged and resuspended in lysis buffer [50 mM Tris–HCl, 500 mM NaCl, and 20 mM imidazole (pH 8.0)]. Bacterial cell suspensions were lysed by ultrasonication and centrifuged. Supernatants were collected and mixed with prewashed nickel-nitrilotriacetic acid HisTrap HP affinity resin (GE Healthcare, USA) at 4 °C overnight for target protein binding, and non-specific binding proteins were washed away with wash buffer (50 mM Tris–HCl, 0.5 M NaCl, and 60 mM imidazole [pH 8.0]). The affinity resins were washed with elution buffer (50 mM Tris–HCl, 0.5 M NaCl, and 250 mM imidazole [pH 8.0]) to collect recombinant histidine-tagged FolP2. Sodium dodecyl sulfate–polyacrylamide gel electrophoresis (SDS-PAGE) was used to verify the target protein.

### Antibody preparation of FolP2

Antibody preparation of FolP2 protein of *M. tuberculosis* H37Ra was performed as previously described^29^.

### Western blot analysis

Western blot analysis was performed as previously described^29^, except that in this case antiserum against the recombinant FolP2 was used.

### Measurement of *p*ABA production levels in different *M. tuberculosis* H37Ra strains using the *E. coli* W3110 Δ*pabB* mutant

*M. tuberculosis* H37Ra, *M. tuberculosis* H37Ra Δ*folC* pMV361::*folC*(I43T), and *M. tuberculosis* H37Ra Δ*folC* pMV361::*folC*(I43A) were cultured in Middlebrook 7H9 with OADC to log-phase and culture filtrates were collected with 0.22 μm filters (Millipore, Merck). Measurement of *p*ABA production levels in these three strains was performed as previously described^29^.

## Supplementary Information

Supplementary Information.

## Data Availability

All data generated or analyzed during this study are included in this article and its Supplementary Information files.
