# Supplementary material for: Mutations of folC cause increased susceptibility to sulfamethoxazole in Mycobacterium tuberculosis
Source: Sci Rep. 2021 Jan 14;11:1352. doi: 10.1038/s41598-020-80213-4 (PMC7809127; doi:10.1038/s41598-020-80213-4)
Supplement: Supplementary file 1 — Supplementary Information. [file 41598_2020_80213_MOESM1_ESM.pdf]

Supplementary Information

Mutations of *folC* cause increased susceptibility to sulfamethoxazole in *Mycobacterium tuberculosis*

Ruiqi Wang[1#], Kun Li[2#], Jifang Yu[1], Jiaoyu Deng[1]*, Yaokai Chen[2]*

1, Key Laboratory of Special Pathogens and Biosafety, Wuhan Institute of Virology, Chinese Academy of Sciences, Wuhan 430071, People's Republic of China

2, Central Laboratory, Chongqing Public Health Medical Center, Chongqing 400036, People's Republic of China

*Corresponding authors. Tel: +86-027-87198676; Fax: +86-027-87199492; E-mail: yaokaichen@hotmail.com, dengjy@wh.iov.cn

[#]These authors contribute equally to the work

Materials and Methods

Genomic DNA extraction from clinical isolates

Colonies of preliminary screened PAS-resistant isolates on L-J solid medium (>1 μg/mL PAS) were transferred into 1.5 mL Eppendorf tubes, suspended in 1 mL deionized water, and centrifuged. Genomic DNA extraction was performed using the HiPure Mycobacterial DNA Kit (Magen, Guangzhou, China). Briefly, the mycobacterial cell pellet was washed twice with deionized water and suspended with 200 μL GTL containing lysozyme (10 μg/mL) and proteinase K (10 μg/mL). The bacterial cells were incubated at 37°C for 20 min, 56°C for 20 min, and 95°C for 20 min and mixed with 600 μL GXP. After centrifugation, the DNA in the supernatant was collected on a silica membrane, washed with GDW, and resuspended in TE buffer.

PAS and SMX susceptibility testing in PAS-resistant clinical isolates with *thyA*, *ribD*, and *folC* mutations

Frozen isolates with *thyA*, *ribD*, and *folC* single mutations were thawed and streaked on 7H10 plates containing 10% Middlebrook OADC (BBL, Becton Dickinson, Sparks, MD, USA) with incubation loops. After 3-4 weeks of incubation at 37°C, single colonies were selected and incubated in Middlebrook 7H9 medium (Difco, Becton Dickinson, Sparks, MD, USA) containing 10% OADC and 0.1% Tween 80 (Merck) for another 3-4 weeks. Afterward, liquid cultures were centrifuged, and cell pellets were washed twice with sterile 0.9% normal saline. Washed cell pellets were resuspended in sterile 0.9% normal saline and adjusted to a McFarland concentration of 1, and then 10-

fold gradient dilutions were performed. Droplets (10 μL) of the 100-fold dilution (~10^5 CFU/mL) series were plated on Petri dishes containing 0.1, 0.2, 0.5, 1, 2, 4, 8, and 16 μg/mL PAS or 5, 10, 20, 50, 100, and 200 μg/mL SMX. The dishes containing bacteria were incubated at 37°C for 3–4 weeks. The MIC was defined as the concentration that inhibits the growth of 99% of tested bacteria.

Bacterial strains, plasmids, and culture conditions

Strains derived from *M. tuberculosis* H37Ra were cultured at 37°C in 7H9 medium consisting of Middlebrook 7H9 broth, 10% (v/v) OADC, 0.5% (v/v) glycerol (Merck), and 0.05% (v/v) Tween 80 or on 7H10 agar medium supplemented with 10% (v/v) OADC and 0.5% (v/v) glycerol. The *E. coli* strain BL21 (DE3) was cultured in LB medium (Merck) at 37°C. Plasmids pET28a (Novagen/Merck, Darmstadt, Germany) and pMV261 were used for the construction of expression plasmids. The gene-specific primers used for the construction of recombinant plasmids are listed in Table S1. Where appropriate, the culture medium was supplemented with hygromycin at 75 μg/mL for *M. tuberculosis* and 150 μg/mL for *E. coli* or kanamycin at 25 μg/mL for mycobacteria and 100 μg/mL for *E. coli*. All lab strains and plasmids are listed in Table S1.

Drug exposure experiments

Bacteria were grown to an OD_{600} of 0.5–1.0 and diluted an OD_{600} of ~0.1 (10^7 CFU/mL) in fresh 7H9 medium with OADC, and 500 μg/mL SMX (10×MIC of wild-type *M. tuberculosis* H37Ra) was used for PAS treatment. Cultures were incubated at 37°C, and

aliquots of samples were taken and plated on 7H10 medium after serial dilutions at days 0, 7, 14, 21, and 28.

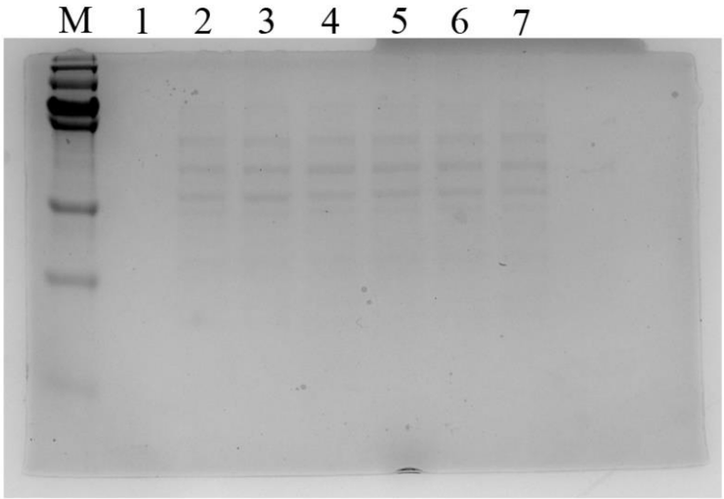

Figure S1. Original figure of SDS-PAGE.

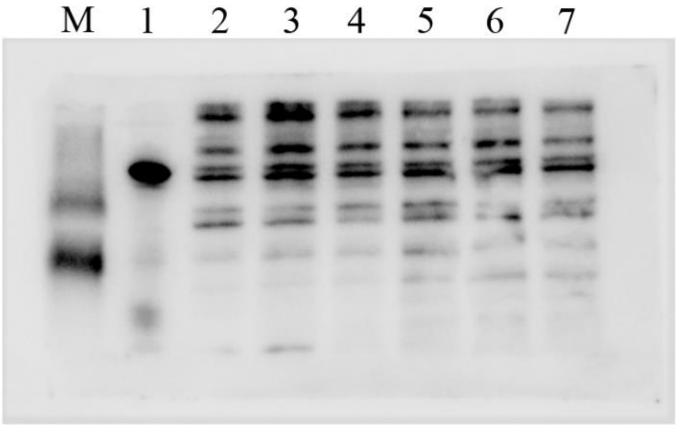

Figure S2. Original figure of Western blot.

Category	Name	Relevant features/sequences	Source
Plasmids			
	pMV261	expression plasmid in *M. tuberculosis*,	Lab stock

		Km^R	
	pET28a::*folP2*	*folP2* expression plasmid, Km^R	This study
Strains			
	H37Ra	*M. tuberculosis* H37Ra	Lab stock
	mc^2155	*M. smegmatis* mc^2155	Lab stock
	H37Ra pMV261	*M. tuberculosis* H37Ra transformed with pMV261	Lab construct
	H37Ra Δ*folP2*	Specialized transduction of strain H37Ra with phAES*folP2*$_{Ra}$	This study
	H37Ra Δ*folC* pMV361::folC(I43T)	*M. tuberculosis* strain H37Ra carrying pMV361-*folC*I43T, chromosomal copy of *folC* deleted	Lab construct
	H37Ra Δ*folC* pMV361::folC(I43A)	*M. tuberculosis* strain H37Ra carrying pMV361-*folC*I43A, chromosomal copy of *folC* deleted	Lab construct
	E. coli BL21 pET21a::*folP2*$_{MTB}$	*E. coli* BL21 transformed with pET21a::*folP2*$_{MTB}$	This study
	E. coli W3110 Δ*pabB*	*E. coli* W3110, chromosomal copy of *pabB* deleted	Lab construct
Primers			
	M*folP2*-F	TTAAGGATCCATGCGTTCAACACC GCCGGCCTCA	This study
	M*folP2*-R	TAATAAGCTTTTATGCGAGTCCTCT CACCGTGCGCGT	This study
	*folP2*koLFP	TTTTTTTTCCATAAATTGG GCCTTTATCAACATCTTCAACGT	This study
	*folP2*koLRP	TTTTTTTTCCATTTCTTGG TGAGGCCGGCGGTGTTGAACGCA C	This study

	*folP2*koRFP	TTTTTTTTCCATAGATTGG ACGCGCACGGTGAGAGGACTCGC ATGA	This study
	*folP2*koRRP	TTTTTTTTCCATCTTTTGG CGAACGCCCAAGTTGACCTGGGC GA	This study
	*folP2*LYZ	CTGCGGTAACGGGCTGCGGCCGG A	This study
	*folP2*RYZ	CACTATCGTCCGGGCCGCCGGGCA	This study
	folP2-qRT-L	CCCGCGAGAAGGTGTTGAT	This study
	folP2-qRT-R	GCAGCCCATGGAAGGTGTT	This study
	sigA-qRT-F	GACGAGATCGGCCAGGTCTAC	This study
	sigA-qRT-R	CGACATAGTCTTGGATTCGATCTG	This study

Table S1. Strains, plasmids, and primers used in this study.

Isolate	sampling time (mm/dd/yyyy)	Sample ID	Gender	Age (years)	Sample type	Species identification	Main associated drug resistance	Drug susceptibility profile
1	10/31/2016	K6457	Male	20	Sputum	MTB	SIR	MDR
2	08/14/2017	K8497	Male	29	Sputum	MTB	SIR	MDR
3	11/14/2016	K6533	Male	79	Sputum	MTB	WT	Pan-susceptible
4	08/26/2016	K5970	Male	46	Sputum	MTB	SIRO	MDR
5	07/24/2017	K8313	Male	51	Sputum	MTB	IR	MDR
6	06/08/2015	K3270	Male	32	Sputum	MTB	WT	Pan-susceptible
7	04/25/2014	E260	Male	40	Sputum	MTB	SIREL	MDR
8	09/24/2017	K8902	Female	21	Pleural fluid	MTB	SIRA	MDR
9	10/14/2016	K6328	Female	31	Sputum	MTB	SIRM	MDR
10	11/07/2016	K6487	Male	28	Sputum	MTB	SIR	MDR
11	10/24/2016	K6407	Male	46	Sputum	MTB	SIR	MDR
12	07/24/2015	K3543	Male	35	Sputum	MTB	SIRA	MDR
13	12/04/2015	K4287	Male	63	Sputum	MTB	SIR	MDR
14	07/29/2016	K5819	Female	33	Sputum	MTB	SILM	Not Defined
15	10/15/2015	E945	Male	52	Other	MTB	SIREL	MDR
16	03/27/2017	F461	Male	51	Sputum	MTB	SIRELAC	XDR
17	08/26/2016	K5999	Male	16	Sputum	MTB	IRO	MDR
18	06/03/2015	K3229	Female	35	Sputum	MTB	SIR	MDR
19	11/20/2015	K4208	Female	36	Sputum	MTB	SIR	MDR

20	09/09/2016	K6079	Male	16	Sputum	MTB	SIRO	MDR
21	09/15/2014	E531	Male	48	Sputum	MTB	SIRELAC	XDR
22	12/29/2016	F330	Female	27	Fiberoptic bronchoscopy lavage fluid	MTB	SIRE	MDR
23	09/25/2015	E940	Male	28	Fiberoptic bronchoscopy lavage fluid	MTB	SREL	Not Defined
24	03/27/2017	F462	Male	52	Sputum	MTB	SIREACL	XDR
25	10/25/2017	K9133	Male	65	Sputum	MTB	SIR	MDR
26	09/30/2014	E576	Female	37	Sputum	MTB	SIRE	MDR
27	01/16/2015	K2481	Female	38	Fiberoptic bronchoscopy lavage fluid	MTB	SIRO	MDR
28	08/24/2017	K8646	Male	21	Sputum	MTB	SIR	MDR
29	06/03/2015	K3211	Female	36	Sputum	MTB	SIR	MDR
30	06/17/2016	K5514	Male	15	Sputum	MTB	SIR	MDR
31	08/04/2016	K5874	Male	45	Sputum	MTB	SIROA	XDR
32	09/28/2017	K8941	Male	27	Sputum	MTB	SIR	MDR
33	03/03/2017	K7234	Female	24	Sputum	MTB	SIR	MDR
34	08/14/2017	K8433	Male	63	Sputum	MTB	SIR	MDR
35	10/15/2015	E944	Male	45	Other	MTB	SIREL	MDR
36	02/17/2015	K2614	Male	17	Sputum	MTB	IRAC	MDR
37	11/27/2015	K4239	Male	40	Sputum	MTB	SIROLAM	XDR
38	11/13/2015	K4160	Male	31	Sputum	MTB	SIR	MDR
39	07/15/2015	K3491	Male	20	Sputum	MTB	SIR	MDR
40	03/16/2016	K4854	Male	21	Cerebrospinal fluid	MTB	SIR	MDR
41	10/18/2016	F241	Male	42	Sputum	MTB	SIREACL	XDR
42	11/28/2016	K6599	Male	30	Sputum	MTB	SIR	MDR
43	06/01/2018	KA792	Male	21	Sputum	MTB	SIR	MDR
44	06/08/2015	K3283	Female	72	Sputum	MTB	SIRO	MDR
45	10/30/2015	E958	Male	23	Other	MTB	SIREACL	XDR
46	05/09/2017	F508	Male	40	Sputum	MTB	SIREACL	XDR
47	12/02/2016	K6640	Female	25	Sputum	MTB	SI	Not Defined
48	09/25/2015	E941	Female	26	Sputum	MTB	SIREL	MDR
49	12/04/2015	K4279	Male	29	Pyogenic fluid	MTB	SIR	MDR
50	11/14/2016	K6545	Female	31	Sputum	MTB	SIRA	MDR
51	07/24/2017	K8315	Male	29	Sputum	MTB	SIRLA	XDR
52	10/15/2014	E578	Male	43	Sputum	MTB	SIREL	MDR
53	07/24/2017	K8301	Female	48	Sputum	MTB	SIRA	MDR
54	06/26/2015	K3361	Male	47	Sputum	MTB	SIREOLM	MDR
55	08/05/2015	E903	Female	23	Other	MTB	SIRELC	XDR
56	01/29/2015	K2543	Female	28	Sputum	MTB	SIRA	MDR
57	03/23/2016	K4913	Male	26	Sputum	MTB	SIRLA	XDR
58	04/14/2017	K7601	Male	44	Sputum	MTB	SIR	MDR
59	12/18/2015	K4385	Male	41	Sputum	MTB	SIREOL	MDR
60	05/26/2016	F122	Male	42	Sputum	MTB	SIEL	Not Defined
61	04/28/2017	K7678	Male	18	Sputum	MTB	SIR	MDR

62	02/02/2016	K4603	Male	50	Sputum	MTB	SIROLM	MDR
63	09/10/2015	E930	Male	41	Sputum	MTB	SREL	Not Defined
64	05/22/2015	K3183	Female	29	Sputum	MTB	SIROAM	XDR
65	10/21/2016	K6361	Male	45	Sputum	MTB	SIRE	MDR
66	04/13/2018	KA387	Male	70	Sputum	MTB	SIREA	MDR
67	03/06/2018	KA045	Male	43	Pyogenic fluid	MTB	SIR	MDR
68	03/20/2018	KA156	Male	64	Sputum	MTB	SIR	MDR
69	05/10/2018	KA636	Female	22	Sputum	MTB	SIR	MDR
70	05/22/2017	K7917	Female	20	Sputum	MTB	SIR	MDR
71	02/06/2017	K7040	Female	27	Sputum	MTB	SIRLM	MDR
72	06/02/2017	K7805	Male	26	Sputum	MTB	SIR	MDR
73	06/12/2017	K7979	Male	29	Fiberoptic bronchoscopy lavage fluid	MTB	SIREA	MDR
74	03/15/2016	F036	Male	43	Sputum	MTB	IREACL	XDR
75	07/09/2015	K3459	Male	58	Sputum	MTB	SIRO	MDR
76	05/06/2016	K5236	Female	36	Sputum	MTB	SIROMA	XDR
77	12/11/2015	K4354	Male	60	Sputum	MTB	SIRA	MDR
78	05/27/2016	K5366	Female	47	Sputum	MTB	SIREOLAM	XDR
79	05/07/2014	E285	Male	49	Sputum	MTB	SIREALC	XDR
80	12/04/2015	K4303	Female	38	Sputum	MTB	SIRA	MDR
81	01/18/2018	F970	Male	36	Other	MTB	SIREL	MDR
82	11/22/2017	F899	Male	23	Sputum	MTB	SIREAL	XDR
83	04/27/2016	F077	Male	24	Sputum	MTB	SIRECL	XDR
84	05/20/2016	K5303	Male	30	Sputum	MTB	SIROL	MDR
85	01/25/2017	F375	Female	41	Sputum	MTB	SIRL	MDR
86	03/23/2018	KA187	Female	27	Sputum	MTB	SIR	MDR
87	12/28/2016	K6842	Female	41	Fiberoptic bronchoscopy lavage fluid	MTB	SIRM	MDR
88	12/12/2016	K6722	Male	38	Sputum	MTB	SIRA	MDR
89	06/01/2018	KA779	Male	55	Sputum	MTB	SIRA	MDR
90	05/25/2018	KA712	Female	44	Sputum	MTB	IR	MDR
91	04/16/2018	KA391	Male	49	Sputum	MTB	IR	MDR
92	04/18/2018	KA440	Female	32	Sputum	MTB	SIR	MDR
93	04/05/2018	G216	Female	50	Sputum	MTB	SIREAL	XDR
94	01/13/2018	K9669	Female	58	Sputum	MTB	SIR	MDR

Table S2. Detailed information of 94 clinical samples used in this study. mm/dd/yyyy: month/day/year; ID: identification; S: streptomycin; I: isoniazid; R: rifampicin; E: ethambutol; O: ofloxacin; L: levofloxacin; A: amikacin; M: moxifloxacin; C: capreomycin; Other, samples which come from feces, blood, or human tissues collected during surgical operations; Not Defined, non-conformity with definitions of pan-susceptible, MDR or XDR.
